# 非小细胞肺癌*AKT1* E17K突变丰度分层及其与*EGFR*共突变的特征分析

**DOI:** 10.3779/j.issn.1009-3419.2026.106.24

**Published:** 2026-08-20

**Authors:** Dan ZHAO, Deyang XIE, Nana ZHANG, Lili ZHANG, Kun LI, Nanying CHE

**Affiliations:** 101149 北京，首都医科大学附属北京胸科医院/北京市结核病胸部肿瘤研究所病理科; Department of Pathology, Beijing Chest Hospital, Capital Medical University/Beijing Tuberculosis and Thoracic Tumor Research Institute, Beijing 101149, China

**Keywords:** 肺肿瘤, *AKT1*, E17K, 突变丰度, *EGFR*, 共突变, 克隆演化, Lung neoplasms, *AKT1*, E17K, Variant allele frequency, *EGFR*, Co-mutation, Clonal evolution

## Abstract

**背景与目的:**

*AKT*丝氨酸/苏氨酸激酶1（*AKT *serine/threonine kinase 1, *AKT1*）E17K是*AKT1*基因最常见的激活突变，在乳腺癌中已被确立为可靶向的驱动事件。然而该突变在非小细胞肺癌（non-small cell lung cancer, NSCLC）中极为罕见，临床意义尚不明确。变异等位基因频率（variant allele frequency, VAF）可在一定程度上反映突变克隆占比，本研究通过分析NSCLC中*AKT1* E17K的VAF与表皮生长因子受体（epidermal growth factor receptor, *EGFR*）等经典驱动基因的共突变关系，探索该突变在肿瘤克隆演化中的潜在意义。

**方法:**

回顾性分析首都医科大学附属北京胸科医院2019年6月至2026年4月肺癌患者下一代测序（next-generation sequencing, NGS）数据，13,003例次检测去重后为11,368例独立患者（含小细胞肺癌），检出*AKT1* E17K阳性35例（总体检出率为0.31%），均为NSCLC，剔除5例外院会诊病例后纳入30例。以VAF连续变量分析为主要分析，以10%为探索性界值进行分组比较。

**结果:**

30例患者中腺癌20例（66.7%），中位随访12个月（范围：1-55个月）。高VAF组（≥10%，17例）*EGFR*共突变率（11.8%, 2/17）低于低VAF组（<10%，13例；46.2%，6/13），校正前*P*为0.049[比值比（odds ratio, OR）=0.156]，Bonferroni校正后差异无统计学意义（*P*=0.246）；*EGFR*共突变阳性组VAF中位数低于阴性组（1.37% *vs* 13.73%, *P*=0.071）；连续变量分析显示VAF与*EGFR*共突变呈负相关趋势（*ρ*=-0.340, *P*=0.066）。Firth校正*Logistic*回归调整组织学类型后，高VAF与*EGFR*共突变减少的关联方向不变，但不再具有统计学差异（OR=0.21, 95%CI: 0.03-1.33, *P*=0.097）。

**结论:**

*AKT1 *E17K VAF与*EGFR*共突变呈负相关趋势，但该关联经多重比较校正及组织学类型调整后均无统计学显著性，可能部分受组织学类型构成影响。本研究结果应作为探索性发现，仍需多中心大样本研究证实。

## Abstract (en)

**Background and objective:**

*AKT* serine/threonine kinase 1 (*AKT1*) E17K, the most common activating mutation of *AKT1*, is an established druggable driver in breast cancer; however, it is exceedingly rare in non-small cell lung cancer (NSCLC), with its clinical significance remaining unclear. Variant allele frequency (VAF) may partially reflect the clonal proportion of a mutation, offering clues to the clonal characteristics of such rare mutations. This study investigated the relationship between* AKT1 *E17K VAF and co-mutation with classic driver genes such as epidermal growth factor receptor (*EGFR*) in NSCLC, aiming to explore the potential role of this mutation in tumor clonal evolution.

**Methods:**

Next-generation sequencing (NGS) data of lung cancer patients at Beijing Chest Hospital, Capital Medical University from June 2019 to April 2026 were retrospectively analyzed. After deduplication of 13,003 tests, 11,368 unique patients (including small cell lung cancer) were identified, among whom 35 harbored *AKT1 *E17K mutations (overall detection rate 0.31%), all being NSCLC; 5 external consultation cases were excluded, leaving 30 cases for analysis. Continuous VAF analysis was designated as the primary analysis, with an exploratory cutoff of 10%.

**Results:**

Of the 30 patients, 20 (66.7%) were adenocarcinoma. Median follow-up was 12 months (range: 1-55 months). The *EGFR* co-mutation rate was lower in the high-VAF group (≥10%, *n*=17) than in the low-VAF group (<10%, *n*=13; 11.8% *vs* 46.2%), reaching nominal significance before correction [*P*=0.049, odds ratio (OR)=0.156] but not after Bonferroni adjustment (adjusted *P*=0.246). The median VAF was lower in the *EGFR* co-mutated group than in the *EGFR* wild-type group (1.37% *vs* 13.73%, *P*=0.071). Continuous analysis showed a negative trend between VAF and *EGFR* co-mutation (*ρ*=-0.340, *P*=0.066). After adjusting for histological type using Firth-corrected *Logistic* regression, the direction of the association remained unchanged but was no longer nominally significant (OR=0.21, 95%CI: 0.03-1.33, *P*=0.097).

**Conclusion:**

*AKT1* E17K VAF showed a negative trend with *EGFR* co-mutation; however, the association did not remain significant after correction for multiple comparisons or adjustment for histological type, and may be partly influenced by histological composition. These findings should be regarded as hypothesis-generating and require validation in larger multi-center cohorts.

原发性支气管肺癌是全球发病率和死亡率最高的恶性肿瘤^[1]^，其中非小细胞肺癌（non-small cell lung cancer, NSCLC）占肺癌的绝大多数，其驱动基因的发现推动了靶向治疗的发展，显著改善了患者的诊疗格局。*AKT*丝氨酸/苏氨酸激酶1（*AKT* serine/threonine kinase 1, *AKT1*）激活是NSCLC中一种罕见的驱动事件^[2,3]^，其编码蛋白是磷脂酰肌醇3-激酶（phosphoinositide 3-kinase, PI3K）/AKT/雷帕霉素机制靶蛋白（mechanistic target of rapamycin, mTOR）信号通路的核心调控节点^[4]^。E17K是AKT1最常见的激活突变^[5]^，该突变可破坏普列克底物蛋白同源结构域（pleckstrin homology, PH）与激酶结构域之间的自抑制相互作用，使*AKT1*不依赖上游信号而持续激活，进而促进肿瘤细胞增殖、存活并诱导耐药^[3]^。一项纳入600余例乳腺癌患者的研究^[6]^显示，*AKT1* E17K在乳腺癌中的总体突变率约为 6.3%，且在低分级肿瘤中富集，而在NSCLC中*AKT1 *E17K突变低于1%，属于少见驱动事件^[2]^。

近年来，针对*AKT1 *E17K的靶向药物卡匹色替（Capivasertib）在乳腺癌等瘤种中已展现出良好疗效^[7,8]^，并于2023年获美国食品药品监督管理局（Food and Drug Administration, FDA）批准联合氟维司群用于伴磷脂酰肌醇-4,5-二磷酸3-激酶催化亚基α（phosphatidylinositol-4,5-bisphosphate 3-kinase catalytic subunit alpha, *PIK3CA*）*/AKT1/*磷酸酶和张力蛋白同源物（phosphatase and tensin homolog, *PTEN*）改变的激素受体阳性（hormone receptor-positive, HR+）/人表皮生长因子受体2阴性（human epidermal growth factor receptor 2-negative, HER2-）晚期乳腺癌的治疗^[7]^。然而，在NSCLC领域*AKT1 *E17K突变的临床意义及最佳治疗策略尚不明确，相关临床证据十分有限^[2,9]^。

突变丰度，即变异等位基因频率（variant allele frequency, VAF）是下一代测序（next-generation sequencing, NGS）检测的核心指标之一。VAF可在一定条件下为突变克隆状态提供间接线索，但受到肿瘤细胞含量、局部拷贝数改变、等位基因不平衡及检测技术等多种因素影响^[10]^。因此，在上述因素相对可比的条件下，较高VAF可能提示突变存在于较高比例的肿瘤细胞中，而仅凭低VAF不能直接判定其为亚克隆事件。突变的克隆状态与肿瘤共突变模式、药物应答及预后密切关联，但目前针对NSCLC中*AKT1* E17K突变的VAF特征与克隆属性研究仍较为缺乏。基于此，本研究依托首都医科大学附属北京胸科医院肺癌患者NGS检测数据，以VAF连续变量为主要分析维度、以≈队列中位数的10%为探索性界值，探讨*AKT1* E17K与经典驱动基因的共突变关系，以期为该少见突变的精准诊疗提供探索性依据。

## 1 材料与方法

### 1.1 病例来源

回顾性分析2019年6月至2026年4月首都医科大学附属北京胸科医院病理科肺癌患者NGS检测数据。研究期间共完成13,003例次检测，去重后为11,368例独立肺癌患者（含小细胞肺癌），其中检出*AKT1* E17K（c.49G>A/p.E17K）突变阳性35例，均为NSCLC；鉴于分母含小细胞肺癌，肺癌总体检出率为0.31%（35/11,368），此检出率非NSCLC专属发生率。剔除5例无法追踪完整临床病史的外院会诊病例（测序质量均合格）后，最终纳入30例连续病例，筛选流程见图1。研究期间检测平台、试剂盒及生信分析流程均未变更。病例纳入标准：（1）经病理确诊为NSCLC；（2）NGS检测示*AKT1* E17K（c.49G>A/p.E17K）突变阳性。本研究经首都医科大学附属北京胸科医院伦理委员会审批（No.2025-008），因为回顾性分析，免除患者知情同意；所有研究流程符合《赫尔辛基宣言》伦理要求。

**图1:**
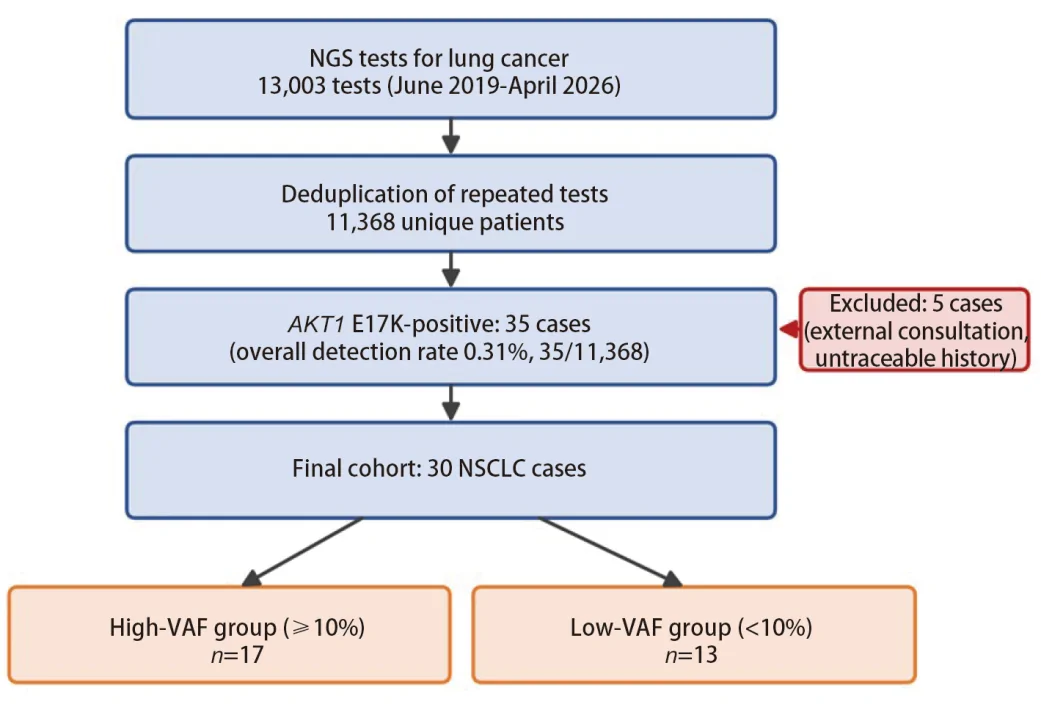
病例筛选流程图

### 1.2 NGS检测方法

#### 1.2.1 DNA与RNA提取

采用核酸提取或纯化试剂（中国天津诺禾致源生物信息科技有限公司）从福尔马林固定石蜡包埋（formalin-fixed paraffin-embedded, FFPE）的组织切片中提取DNA和RNA。使用Qubit 3.0荧光计（美国Life Technologies公司）对提取所得DNA和RNA的浓度进行定量分析，要求DNA总量>10 ng，RNA总量>30 ng。

#### 1.2.2 NGS文库构建与测序

本研究同时构建DNA和RNA文库：RNA文库用于检测基因融合，DNA文库用于检测点突变、插入缺失和拷贝数扩增。DNA和RNA文库采用人表皮生长因子受体（epidermal growth factor receptor, *EGFR*）、Kirsten大鼠肉瘤病毒癌基因同源物（Kirsten rat sarcoma viral oncogene homolog, *KRAS*）、V-raf鼠肉瘤病毒癌基因同源体B1（v-raf murine sarcoma viral oncogene homolog B1, *BRAF*）、*PIK3CA*、间变性淋巴瘤激酶（anaplastic lymphoma kinase, *ALK*）和*ROS*原癌基因1受体酪氨酸激酶（*ROS* proto-oncogene 1, receptor tyrosine kinase, *ROS1*）基因突变检测试剂盒（半导体测序法）（中国天津诺禾致源生物信息科技有限公司）按制造商说明书构建。该检测panel可覆盖60个肺癌相关基因的重要外显子区和内含子区使用Qubit 3.0荧光计和ABI 7500实时荧光定量聚合酶链反应（polymerase chain reaction, PCR）系统对构建的文库进行定量。测序工作在DA8600高通量测序仪（中国广州达安基因股份有限公司）完成。

#### 1.2.3 测序质控指标

DNA文库平均测序深度>2000×；RNA文库序列数>60,000条。本研究采用扩增子法建库，对低水平扩增[拷贝数变异（copy number variation, CNV）<2倍]的灵敏度略低于捕获法，因此仅纳入CNV≥2倍的扩增作为阳性。DNA样本目标区域覆盖度≥99%，*AKT1 *E17K位点最低检测下限（limit of detection, LoD）为1%，融合基因检测灵敏度为1%。需要指出的是，本研究未根据肿瘤细胞纯度计算癌细胞分数（cancer cell fraction, CCF），VAF的动态变化可能受到样本间肿瘤细胞纯度差异的影响，因此VAF结果仅供探索性参考。同一患者多次检测者取首次治疗前的检测结果纳入分析。

#### 1.2.4 生物信息学分析

原始测序数据经SAMtools转换格式、排序及索引后，使用TMAP比对工具与人类参考基因组（GRCh37/hg19）进行比对。采用Torrent Variant Caller（TVC）和MuSCAN两种工具互补检测单核苷酸变异（single nucleotide variants, SNVs）及小片段插入缺失（insertions and deletions, InDels），经ANNOVAR和SnpEff进行功能注释后，筛选具有临床意义的突变。

### 1.3 数据处理与统计分析

#### 1.3.1 主要分析

将*AKT1* E17K VAF作为连续变量，采用*Mann-Whitney U*检验（秩和检验）比较*EGFR*共突变阳性与阴性组的VAF分布，并采用*Spearman*秩相关分析评估VAF与*EGFR*共突变状态的关联强度。探索性分组分析：以本队列VAF中位数（10.99%）为参照，以10%作为探索性界值将患者划分为高VAF组（≥10%）与低VAF组（<10%），组间分类变量的比较使用*Fisher*精确检验。其中吸烟状态、组织学类型、分化程度及临床分期均为2×3列联表，采用*Freeman-Halton*精确检验（*Fisher*精确检验的r×c扩展）。需强调的是，该界值为基于本队列数据分布的事后选择，仅用于探索性分层，不具有确证性意义。

#### 1.3.2 混杂因素控制与敏感性分析

鉴于组织学类型与EGFR突变谱密切相关（肺鳞癌中典型*EGFR*驱动改变较少见），本研究采用以下敏感性分析与校正策略：（1）将腺癌亚组分析作为关键敏感性分析，并采用*Kruskal-Wallis*检验比较不同腺癌亚型间VAF分布（观察性分析）；（2）采用Firth校正*Logistic*回归（惩罚最大似然法，可降低小样本偏倚并处理完全或近完全分离问题^[11,12]^），以*EGFR*共突变状态为因变量，分别拟合两个模型：模型一纳入VAF分组（高/低）与组织学类型（腺癌 vs 非腺癌），模型二纳入VAF连续变量与组织学类型；（3）采用5%、10%、15%、20%、25%、30%多个界值进行敏感性分析，以评估分组结果对界值选择的依赖程度。本队列*EGFR*共突变事件仅8例，为降低小样本模型过拟合和估计不稳定风险，本研究仅选择组织学类型这一最重要的潜在混杂因素进行调整，未进一步纳入临床分期、吸烟状态、取材方式及肿瘤纯度等变量。此外，还对*EGFR*、*KRAS*及*BRAF*（均为肺癌经典驱动基因）的联合阳性状态（3类基因任一突变阳性即为联合阳性）作为复合终点进行了探索性分析。组间比较的比值比（odds ratio, OR）为样本OR，其95%可信区间（confidence interval, CI）基于对数尺度Wald法计算；存在零单元格时（*PIK3CA*）OR经*Haldane-Anscombe*校正；组间比较采用*Fisher*精确检验。

鉴于共突变事件数差异较大，为避免极低事件数导致的估计不稳定，本研究预先规定：仅对共突变事件数≥2例的5个基因[*EGFR*、*BRAF*、间质-上皮细胞转化因子（mesenchymal-epithelial transition factor, *MET*）、肿瘤蛋白p53（tumor protein p53, *TP53*）、*PIK3CA*]进行正式组间推断并纳入多重比较校正；*KRAS*与*PTEN*（事件数均仅1例）仅作描述性展示，不进行正式假设检验。

鉴于本研究进行了多次独立比较（*EGFR*、*BRAF*、*MET*、*TP53*、*PIK3CA*共突变率的组间比较），进行Bonferroni多重比较校正，校正后显著性水准α=0.05/5=0.01，仅适用于上述5个基因共突变率的组间比较。经典驱动基因联合终点及不同VAF界值的分组比较均作为探索性分析，不纳入正式假设检验，故不参与多重比较校正。未预先进行样本量估算。缺失数据采用可用病例分析（available-case analysis），未进行数据插补。

统计分析采用SPSS 26.0软件（IBM, USA）及Python（SciPy、statsmodels库）完成，Firth校正*Logistic*回归采用Python基于惩罚最大似然法（Jeffreys先验）^[11,12]^编程实现，其结果经独立复算验证，使用双侧检验，*P*<0.05为差异具有统计学意义。

## 2 结果

### 2.1 病例筛选与临床病理特征

30例患者中，男性17例（56.7%），女性13例（43.3%）；年龄范围29-79岁，中位年龄67岁。病理类型以腺癌为主（20例，66.7%），其余为鳞癌8例（26.7%）、腺鳞癌1例（3.3%）、梭形细胞癌1例（3.3%）。吸烟状态：从不吸烟22例（73.3%），既往或当前吸烟7例（23.3%），不详1例（3.3%）。临床分期：IV期16例（53.3%），I-II期8例（26.7%），III期4例（13.3%），无法评估2例（6.7%）。中位随访时间为12个月（范围：1-55个月），末次随访时间为2026年4月，存活27例（90.0%），死亡2例（6.7%），失访1例（3.3%）。鉴于死亡事件仅2例，未进行生存分析。

*AKT1* VAF范围为1.02%-82.91%，中位数为10.99%。结合本队列VAF中位数水平，以10%作为探索性界值，将患者分为高VAF组（≥10%, *n*=17, 56.7%）与低VAF组（<10%, *n*=13, 43.3%）。组间基线分析（表1）显示，两组患者在年龄、性别、吸烟状态、分化程度、临床分期及随访时间方面的差异均无统计学意义（*P*>0.05）；组织学类型构成存在一定组间差异，高VAF组鳞癌占比为35.3%（6/17），低VAF组为15.4%（2/13），但两组差异无统计学意义，考虑到NSCLC不同组织学亚型的驱动基因谱存在固有差异（肺鳞癌极少发生*EGFR*驱动改变），可能对共突变分析产生混杂影响，故本研究将组织学类型纳入敏感性分析与校正模型（见2.4）。

**表1:** 30例AKT1 E17K突变型NSCLC临床病理特征及VAF分层比较

Characteristic	Overall (*n*=30)	High VAF (≥10%, *n*=17)	Low VAF (<10%, *n*=13)	*P*
Age (yr), median (range)	67 (29-79)	66 (29-79)	67 (44-75)	0.917^a^
Sex, n (%)				0.283^b^
Male	17 (56.7)	8 (47.1)	9 (69.2)	
Female	13 (43.3)	9 (52.9)	4 (30.8)	
Smoking status, n (%)				0.811^c^
Never smoker	22 (73.3)	13 (76.5)	9 (69.2)	
Former/current smoker	7 (23.3)	3 (17.6)	4 (30.8)	
Unknown	1 (3.3)	1 (5.9)	0 (0.0)	
Histological type, n (%)				0.597^c^
Adenocarcinoma	20 (66.7)	10 (58.8)	10 (76.9)	
Squamous cell carcinoma	8 (26.7)	6 (35.3)	2 (15.4)	
Others	2 (6.7)	1 (5.9)	1 (7.7)	
Tumor differentiation, n (%)				0.646^c^
Well/moderately differentiated	8 (26.7)	4 (23.5)	4 (30.8)	
Poorly differentiated	12 (40.0)	6 (35.3)	6 (46.1)	
Not specified	10 (33.3)	7 (41.2)	3 (23.1)	
TNM stage, n (%)				0.112^c^
Stage I-II	8 (26.7)	2 (11.8)	6 (46.2)	
Stage III	4 (13.3)	3 (17.6)	1 (7.7)	
Stage IV	16 (53.3)	11 (64.7)	5 (38.5)	
Stage not assessable	2 (6.7)	1 (5.9)	1 (7.7)	
Survival status, n (%)				0.710^b^
Alive	27 (90.0)	16 (94.1)	11 (84.6)	
Dead	2 (6.7)	1 (5.9)	1 (7.7)	
Lost to follow-up	1 (3.3)	0 (0.0)	1 (7.7)	
Follow-up (mon), median	12	11	12	0.792^a^
AKT1 VAF, median	10.99%	22.77%	1.38%	<0.001^a^

^a^Mann-Whitney U test; ^b^Fisher’s exact test; ^c^Freeman-Halton exact test on the 2×3 table of assessable cases; two cases (6.7%) with unassessable stage were excluded from this comparison. The distribution of histological types differed between the high- and low-VAF groups (squamous cell carcinoma: 35.3% vs 15.4%). Although this difference did not reach statistical significance, histological type was incorporated into sensitivity analyses and adjusted models as a potential confounder. AKT1: AKT serine/threonine kinase 1; NSCLC: non-small cell lung cancer; TNM: tumor-node-metastasis; VAF: variant allele frequency.

### 2.2 VAF连续变量与*EGFR*共突变的关系（主要分析）

将*AKT1* E17K的VAF作为连续变量进行分析：*EGFR*共突变阳性组的VAF中位数为1.37%（范围：1.05%-46.82%），阴性组为13.73%（范围：1.02%-82.91%）；*Mann-Whitney U*检验显示，两组间差异未达统计学意义（*Z*=-1.80, *P*=0.071）。*Spearman*秩相关分析显示，*AKT1 *VAF与*EGFR*共突变呈负相关趋势（*ρ*=-0.340, *P*=0.066），亦未达统计学意义。30例患者VAF呈右偏态分布：<5%者12例（40.0%），≥5%且<10%者1例（3.3%），≥10%且<15%者5例（16.7%），≥15%且<20%者3例（10.0%），≥20%且<25%者1例（3.3%），≥25%且<30%者1例（3.3%），≥30%者7例（23.3%）。8例*EGFR*共突变中5例（62.5%）VAF<5%，仅2例（25.0%）VAF≥10%，提示*EGFR*共突变集中于低VAF区间。

为进一步探索*AKT1* VAF与驱动基因共突变的普遍关联，将*EGFR*、*KRAS*、*BRAF*合并为“经典驱动基因联合终点”进行探索性分析：联合阳性组（*n*=14）与阴性组（*n*=16）的VAF中位数分别为9.46%和17.03%，组间差异无统计学意义（*P*=0.170）；*Spearman*秩相关分析亦未显示显著关联（*ρ*=-0.259, *P*=0.168）。

### 2.3 共突变谱特征与VAF分层（探索性界值）

在30例患者中共检测到*TP53*突变11例（36.7%）、*EGFR*突变8例（26.7%）、*BRAF*突变5例（16.7%）、*MET*基因改变3例[10.0%,含扩增1例（CNV为2.025倍）、外显子14剪接变异1例、点突变1例]、*KRAS*突变1例（3.3%）、*PIK3CA*突变2例（6.7%）及*PTEN*突变1例（3.3%）。PI3K/AKT/mTOR通路内其他基因（*PIK3CA*、*PTEN*、*mTOR*）共突变患者合计3例（10.0%）。按*AKT1* VAF从高到低排序，8例*EGFR*共突变中5例位于VAF最低的12例（<5%）之内，高VAF组（≥10%，17例）仅2例；而TP53突变（11例）在高、低VAF两组分别为7例与4例（*P*=0.708），遍布全VAF范围，提示其与*AKT1*克隆丰度无明显关联。

本队列*EGFR*突变率（26.7%）低于PIONEER研究^[13]^报道的亚洲晚期肺腺癌人群*EGFR*突变率（51.4%）。由于不同研究在人群特征、病理类型构成、疾病分期、检测平台及入组时间上存在明显差异，该比较仅作描述性参考，不支持任何因果推断。以10%为探索性界值的VAF分层分析显示，高VAF组的*EGFR*共突变率（11.8%, 2/17）低于低VAF组（46.2%, 6/13），校正前差异具有统计学意义（*P*=0.049, OR=0.156），但经Bonferroni多重校正后未达显著性水准（校正后*P*=0.246）。*BRAF*、*MET*、*TP53*等伴随分子改变在两组间均无显著差异（*P*>0.05，表2）。需要说明的是，仅*EGFR*在校正前有显著差异而其他基因无显著差异，并不能证明不同基因与VAF的关联强度存在差异；*KRAS*、*PTEN*均为1例，仅作描述性展示；*PIK3CA*虽纳入预设正式比较，但事件数仅2例，统计估计不稳定，表2中相应统计量仅供探索性参考。本研究样本量不足以评估或比较不同基因事件与VAF的关联强度。

**表2:** VAF分层与主要伴随基因改变的关联

Gene	Overall (*n*=30)	High VAF (*n*=17)	Low VAF (*n*=13)	*P*	OR (95%CI)	Adjusted *P*
TP53	11 (36.7%)	7 (41.2%)	4 (30.8%)	0.708	1.575 (0.343-7.224)	>0.999
EGFR	8 (26.7%)	2 (11.8%)	6 (46.2%)	0.049	0.156 (0.025-0.974)	0.246
BRAF	5 (16.7%)	4 (23.5%)	1 (7.7%)	0.355	3.692 (0.360-37.860)	>0.999
MET	3 (10.0%)	2 (11.8%)	1 (7.7%)	1.000	1.600 (0.129-19.840)	>0.999
KRAS	1 (3.3%)	1 (5.9%)	0 (0.0%)	-	-	-
PIK3CA	2 (6.7%)	0 (0.0%)	2 (15.4%)	0.179	0.131 (0.006-2.995)	0.895
PTEN	1 (3.3%)	0 (0.0%)	1 (7.7%)	-	-	-
EGFR+KRAS+BRAF	14 (46.7%)	7 (41.2%)	7 (53.8%)	0.713	0.600 (0.140-2.575)	-

Formal between-group comparisons for TP53, EGFR, BRAF, MET and PIK3CA were performed using Fisher’s exact test. KRAS and PTEN, each observed in only one case, were presented descriptively without formal hypothesis testing. The EGFR+KRAS+BRAF composite endpoint was analyzed exploratorily. For EGFR, the unadjusted P value was 0.049, which did not remain significant after Bonferroni correction for the five prespecified gene-level comparisons (adjusted P=0.246). The OR for PIK3CA was calculated using the Haldane-Anscombe correction for the zero cell. Percentages in parentheses are within-group proportions (n/N); because one patient could harbor multiple gene alterations, the percentages across rows do not sum to 100%. Adjusted P values were obtained by Bonferroni correction (multiplied by 5) across the five prespecified gene-level comparisons and were capped at 1. TP53: tumor protein p53; EGFR: epidermal growth factor receptor; BRAF: B-Raf proto-oncogene, serine/threonine kinase; MET: mesenchymal-epithelial transition factor; KRAS: Kirsten rat sarcoma viral oncogene homolog; PIK3CA: phosphatidylinositol-4,5-bisphosphate 3-kinase catalytic subunit alpha; PTEN: phosphatase and tensin homolog; OR: odds ratio; CI: confidence interval.

### 2.4 敏感性分析与混杂因素控制

为评估VAF与*EGFR*共突变关联的稳健性，本研究从组织学混杂控制与界值选择稳定性两方面进行敏感性分析。

#### 2.4.1 组织学类型分层（关键敏感性分析）

在腺癌亚组（*n*=20）中，高VAF组*EGFR*共突变率为20.0%（2/10），低VAF组为60.0%（6/10），差异无统计学意义（*P*=0.170，*Fisher*精确检验），效应方向与总体分析一致；鳞癌亚组（*n*=8）中未观察到*EGFR*共突变（0/8），与肺鳞癌较少发生*EGFR*驱动改变的已知背景一致；梭形细胞癌与腺鳞癌各1例，因例数过少未单独分析，在校正模型中归入非腺癌组。这提示总体分析中观察到的关联可能部分受两组组织学类型构成差异的影响。此外，按腺癌亚型的观察性分析显示：贴壁型（*n*=2）均表现为低VAF且伴*EGFR*共突变；实体型（*n*=3）VAF最高（中位39.37%），*EGFR*共突变率为33.3%（1/3）；按6种腺癌亚型比较，VAF分布差异无统计学意义（*Kruskal-Wallis H*=5.231, *P*=0.388）。

#### 2.4.2 Firth校正*Logistic*回归分析

针对本队列事件数较少的特点，采用Firth校正*Logistic*回归控制混杂。调整组织学类型（腺癌 *vs* 非腺癌）后，模型一（VAF分组）中高VAF组与*EGFR*共突变减少的关联方向不变（OR=0.21, 95%CI: 0.03-1.33），但无统计学差异（*P*=0.097），腺癌较非腺癌的OR为12.58（95%CI: 0.55-288.63, *P*=0.113）；模型二（VAF连续变量）中VAF每升高10%，*EGFR*共突变的OR为0.85（95%CI: 0.53-1.35, *P*=0.486），腺癌较非腺癌的OR为11.41（95%CI: 0.57-227.20, *P*=0.111）。

#### 2.4.3 多界值敏感性分析

分别采用5%、10%、15%、20%、25%、30%界值进行分组比较，仅10%界值时*EGFR*共突变率的组间差异校正前达到统计学意义（*P*=0.049），其余界值差异均无统计学意义（*P*=0.210-0.638，表3），提示分组分析结果对界值选择较为敏感，多界值尝试后单一界值的统计学差异可能反映多重比较导致的偶然发现。

**表3:** 不同VAF界值的敏感性分析

Cut off value	High VAF group EGFR+	Low VAF group EGFR+	*P*	OR
5%	16.7% (3/18)	41.7% (5/12)	0.210	0.280
10%	11.8% (2/17)	46.2% (6/13)	0.049	0.156
15%	16.7% (2/12)	33.3% (6/18)	0.419	0.400
20%	11.1% (1/9)	33.3% (7/21)	0.374	0.250
25%	12.5% (1/8)	31.8% (7/22)	0.391	0.306
30%	14.3% (1/7)	30.4% (7/23)	0.638	0.381

All comparisons were performed using Fisher's exact test. Only the 10% cutoff yielded a nominally significant between-group difference before correction (P=0.049); all other cutoffs were non-significant (P=0.210-0.638), indicating that the stratified results are sensitive to cutoff selection. Percentages are within-group EGFR co-mutation rates at each cutoff (n/N), with N being the number of patients in the corresponding VAF group.

## 3 讨论

*AKT1* E17K突变在肺癌中属于罕见事件，其在肿瘤发生发展中的生物学作用及临床意义尚未完全阐明。本研究从11,368例肺癌NGS数据中筛选出30例携带*AKT1* E17K突变的NSCLC患者，各分析一致提示VAF与*EGFR*共突变呈负相关趋势。一种可能的解释是，在部分病例中较高*AKT1 *E17K VAF可能反映该变异存在于相对较高比例的肿瘤细胞中；而*EGFR*共突变病例中观察到的较低*AKT1 *E17K VAF，则提示其克隆构成可能与*EGFR*野生型病例不同。既往研究^[14,15]^表明，肿瘤驱动事件及相关伴随改变可呈现不同的克隆或亚克隆分布。然而，由于本研究未进行肿瘤纯度、局部拷贝数及CCF校正，因此不能仅依据VAF将*AKT1* E17K直接界定为克隆性或亚克隆性事件。观察到的关联还可能部分由高、低VAF组间组织学类型构成差异所致（高VAF组鳞癌占比更高，而鳞癌极少发生*EGFR*突变）；既往Do等^[2]^对219例NSCLC的筛查中，唯一检出的E17K阳性病例即为鳞癌（141例腺癌均为阴性），与本研究高VAF组鳞癌占比偏高的观察一致。故上述解释仅属探索性假说。

本研究的核心发现处于统计学显著性的临界水平，且经多重校正与混杂控制后差异无统计学意义：以10%为探索性界值的分组分析校正前*P*=0.049，Bonferroni校正后*P*=0.246；主要分析（VAF连续变量）中*Mann-Whitney U*检验*P*=0.071、*Spearman*相关*P*=0.066；Firth校正*Logistic*回归调整组织学类型后，分组关联亦不再具有统计学差异（*P*=0.097）。因此，本研究结果应被视为"探索性发现"（hypothesis-generating），而非确证性证据。解读时应重点关注效应量与方向的一致性（各分析中*EGFR*共突变均集中于低VAF组），而非依赖单一*P*值。

关于VAF分层界值，本研究以队列中位数（10.99%）为参照设定10%探索性界值。多界值敏感性分析显示，仅10%界值时组间差异校正前达到显著性，其余界值均无显著差异（*P*=0.210-0.638），该结果对界值选择敏感，可能系多重尝试导致的偶然发现，不能据此认为10%具有特殊生物学意义。此外，本研究仅在*EGFR*中观察到与VAF的关联趋势，由于其他基因事件数极少，不足以比较不同基因事件与VAF的关联强度，是否存在基因特异性有待大样本研究验证。

与乳腺癌中AKT1 E17K的研究相比，现有证据已较为深入。Bleeker等^[5]^于2008年采用PCR扩增联合Sanger直接测序法，在764例实体瘤中筛查*AKT1* E17K突变，是较早开展该位点泛实体瘤筛查的研究之一。该突变在乳腺癌中的发生率约为6.3%^[6]^。NCI-MATCH子研究EAY131-Y^[8]^纳入了35例携带*AKT1* E17K突变的晚期实体瘤患者（以乳腺癌和妇科肿瘤为主），采用泛AKT抑制剂Capivasertib治疗，客观缓解率达28.6%，中位无进展生存期为5.5个月；相关研究^[16]^支持*AKT1* E17K作为可靶向分子异常的临床意义，但尚不足以将其在不同瘤种中一概视为已经确证的疗效预测标志物。2023年，基于CAPItello-291试验结果^[7]^，美国FDA批准了Capivasertib联合氟维司群用于HR+/HER2-晚期乳腺癌（至少携带1个*PIK3CA*/*AKT1*/*PTEN*突变）的治疗。

与乳腺癌不同，肺癌中*AKT1* E17K突变极为罕见（发生率<1%^[2]^）。既往乳腺癌研究^[6]^显示，*AKT1* E17K可与其他致癌改变共存，但亦存在仅检出*AKT1* E17K作为已知驱动改变的病例；同时，*PIK3CA*改变在*AKT1 *E17K突变肿瘤中相对少见。因此，本研究观察到的*AKT1* E17K VAF与*EGFR*共突变关系，可作为不同分子背景下*AKT1*克隆特征可能存在差异的探索性线索，但不同癌种之间不宜直接类比。鉴于本研究为探索性分析且存在组织学构成差异等混杂，*AKT1* E17K的克隆角色及其与驱动基因背景的关系仍有待进一步研究。

PI3K/AKT/mTOR通路的双重激活在本组并不常见，本组病例中该通路其他组分（*PIK3CA*、*PTEN*、*mTOR*）的共突变仅3例（10.0%），且未显示与VAF分层存在显著关联。值得注意的是，既往硬化性肺细胞瘤研究^[17]^发现*AKT1*突变可作为较为突出的分子事件。虽然该肿瘤与NSCLC在生物学行为和分子背景上存在明显差异，不能直接外推至NSCLC，但这一现象从侧面提示*AKT1*异常在特定肺肿瘤中可能具有相对独立的驱动作用。本研究中*PIK3CA*、*PTEN*及*mTOR*共突变事件较少，其是否反映通路内互斥或功能冗余，尚需更大样本及功能研究验证。此外，腺癌亚型层面的观察性分析显示不同亚型间VAF分布存在差异趋势，但仅为描述性观察，不构成统计推断。

从治疗角度看，目前针对PI3K/AKT/mTOR通路的靶向药物在NSCLC中的研发进展缓慢^[9]^。后续随机II期研究提示该通路抑制剂的疗效与肿瘤分子背景密切关联：PAKT试验^[18]^Capivasertib联合紫杉醇一线治疗转移性三阴性乳腺癌延长了总生存[中位总生存期（overall survival, OS）：19.1 *vs* 12.6个月]，且获益在*PIK3CA*/*AKT1*/*PTEN*改变亚组中更为突出；LOTUS试验^[19]^同样显示，携带上述通路改变的患者从Ipatasertib联合紫杉醇中获益更明显。AKT抑制可能解除对受体酪氨酸激酶表达的反馈性抑制，为联合策略提供潜在的机制依据。现有*AKT1*突变实体瘤研究^[16,20]^支持*AKT1* E17K的可靶向性，但尚未基于*AKT1* E17K VAF在NSCLC中验证治疗分层价值。因此，本研究观察到的VAF与*EGFR*共突变负相关趋势仅可作为未来临床试验分层假设的生成线索：高VAF且*EGFR*野生型患者是否更可能从AKT抑制剂单药获益，以及低VAF伴*EGFR*共突变者是否存在联合治疗价值，均需前瞻性试验验证，目前不能据此指导临床实践。

理论上AKT信号通路可能通过上调程序性死亡配体1（programmed death-ligand 1, PD-L1）表达参与免疫逃逸，本研究中*TP53*共突变率为36.7%，但本研究无免疫治疗反应数据，上述内容仅为理论推测，不宜直接指导临床决策。在本组中，共突变最常见的是*TP53*（11例，36.7%；高、低VAF组分别为7例与4例，*P*=0.708），遍布全VAF范围，与*AKT1*克隆丰度无明显关联。本研究未按*TP53*位点或功能类型分层，样本量有限，故不宜对其治疗或预后意义作进一步推断。

本研究存在以下局限性：（1）缺乏同期匹配的*AKT1*野生型NSCLC对照组，无法直接比较*EGFR*突变发生率，与文献报道率的比较仅作描述性参考；（2）存在组织学类型混杂风险，高VAF组鳞癌占比更高而鳞癌极少发生*EGFR*突变，腺癌亚组分析与Firth校正回归均提示总体关联可能部分受组织学构成影响；（3）10%界值为基于本队列中位数的事后选择，多界值敏感性分析显示分组结果对界值选择敏感，该界值不具有确证性意义；（4）协变量调整受限，本队列*EGFR*事件仅8例，为降低小样本模型过拟合和估计不稳定风险，仅选择组织学类型这一最重要的潜在混杂因素进行调整，未能同时纳入临床分期、吸烟状态、取材方式等因素，且未常规检测肿瘤纯度；（5）外院会诊病例剔除可能引入选择偏倚；（6）为单中心回顾性设计，样本量较小（*n*=30），统计效能有限，结果属于探索性假说，需多中心大样本验证；（7）实测VAF受肿瘤纯度、CNV及检测技术敏感度的综合影响，不完全等同于突变在肿瘤细胞中的真实频率；（8）随访数据来源于电子病历系统，部分患者随访时间较短（中位12个月），全组仅2例死亡事件，无法评估VAF分层与长期生存的关系，也未系统记录治疗暴露信息；（9）PCR-based Panel对基因融合和CNV的检测能力弱于大Panel，可能存在漏检；（10）未进行功能实验，无法直接证实VAF差异对应的生物学活性差异。未来研究可通过配对正常样本和全基因组CNV分析进行精确的CCF校正，并前瞻性验证VAF分层与AKT抑制剂疗效的关联。

在*AKT1* E17K突变型NSCLC中，VAF与*EGFR*共突变呈负相关趋势，但经多重比较校正及组织学类型调整后均无统计学显著性，该关联可能部分受组织学类型构成影响，且分组结果对界值选择敏感。因此，本研究结果应作为探索性发现：高VAF可能提示*AKT1* E17K在部分肿瘤中具有相对较高的克隆占比，而低VAF者更常见于*EGFR*共突变背景，这一假说需多中心、大样本并充分控制组织学类型等混杂因素的前瞻性研究验证，目前尚不能指导临床治疗决策。
